# Characterization of *Bacillus amyloliquefaciens* BA-4 and its biocontrol potential against *Fusarium*-related apple replant disease

**DOI:** 10.3389/fpls.2024.1370440

**Published:** 2024-04-19

**Authors:** Bo Li, Xiaoxing He, Saiya Guo, Dongxu Li, Yanan Wang, Xianglong Meng, Pengbo Dai, Tongle Hu, Keqiang Cao, Shutong Wang

**Affiliations:** ^1^ College of Plant Protection, State Key Laboratory of North China Crop Improvement and Regulation, Hebei Agricultural University, Baoding, China; ^2^ Institute of Agricultural Information and Economics, Hebei Academy of Agriculture and Forestry Sciences, Shijiazhuang, Hebei, China

**Keywords:** *Bacillus amyloliquefaciens*, *Fusarium*, apple replant disease, biocontrol, colonization

## Abstract

Apple replant disease (ARD), caused by *Fusarium* pathogens, is a formidable threat to the renewal of apple varieties in China, necessitating the development of effective and sustainable control strategies. In this study, the bacterial strain BA-4 was isolated from the rhizosphere soil of healthy apple trees in a replanted orchard, demonstrating a broad-spectrum antifungal activity against five crucial apple fungal pathogens. Based on its morphology, physiological and biochemical traits, utilization of carbon sources, and Gram stain, strain BA-4 was tentatively identified as *Bacillus amyloliquefaciens*. Phylogenetic analysis using *16S rDNA* and *gyrB* genes conclusively identified BA-4 as *B. amyloliquefaciens*. In-depth investigations into *B. amyloliquefaciens* BA-4 revealed that the strain possesses the capacity to could secrete cell wall degrading enzymes (protease and cellulase), produce molecules analogous to indole-3-acetic acid (IAA) and siderophores, and solubilize phosphorus and potassium. The diverse attributes observed in *B. amyloliquefaciens* BA-4 underscore its potential as a versatile microorganism with multifaceted benefits for both plant well-being and soil fertility. The extracellular metabolites produced by BA-4 displayed a robust inhibitory effect on *Fusarium* hyphal growth and spore germination, inducing irregular swelling, atrophy, and abnormal branching of fungal hyphae. In greenhouse experiments, BA-4 markedly reduced the disease index of *Fusarium*-related ARD, exhibiting protective and therapeutic efficiencies exceeding 80% and 50%, respectively. Moreover, BA-4 demonstrated plant-promoting abilities on both bean and *Malus robusta* Rehd. (MR) seedlings, leading to increased plant height and primary root length. Field experiments further validated the biocontrol effectiveness of BA-4, demonstrating its ability to mitigate ARD symptoms in MR seedlings with a notable 33.34% reduction in mortality rate and improved biomass. Additionally, BA-4 demonstrates robust and stable colonization capabilities in apple rhizosphere soil, particularly within the 10-20 cm soil layer, which indicates that it has long-term effectiveness potential in field conditions. Overall, *B. amyloliquefaciens* BA-4 emerges as a promising biocontrol agent with broad-spectrum antagonistic capabilities, positive effects on plant growth, and strong colonization abilities for the sustainable management of ARD in apple cultivation.

## 1 Introduction

Apple replant disease (ARD) represents a significant soil-related challenge impacting apple cultivation on a global scale (Kanfra et al., 2022; Somera and Mazzola, 2022). It poses a substantial threat to the apple industry, especially in regions where the necessity to replant apple trees or replace varieties arises. ARD predominantly affects newly replanted apple trees, resulting in stunted growth, heightened susceptibility to diseases, root discoloration, root tip necrosis, diminished root biomass, and potential plant mortality during the initial growth season (Kanfra et al., 2022). Furthermore, even mature apple trees suffer from decreased yield and fruit quality when affected by ARD, with severe cases resulting in tree mortality (Mahnkopp et al., 2018; Cavael et al., 2021). ARD is instigated by the accumulation of phenolic compounds or phytotoxins in the roots affected by the disease, coupled with deleterious soil pathogens, notably fungi (*Fusarium*, *Rhizoctonia* and *Cylindrocarpon*) and oomycetes (*Pythium* and *Phytophthora*) (Kelderer et al., 2012; Manici et al., 2018; Wang et al., 2022a). Among the pathogens associated with ARD, recent studies have increasingly highlighted that *Fusarium* species are one of the primary contributors to the occurrence of ARD in China (Wang et al., 2022b; Duan et al., 2023).

*Fusarium* spp. constitute a prevalent group of soilborne pathogens, encompassing various species linked to ARD, such as *F. oxysporum*, *F. moniliforme*, *F. proliferatum*, and *F. solani* (Xiang et al., 2021b; Liu et al., 2022; Ajeethan et al., 2023).These pathogens exhibit remarkable resilience in the soil, persisting as chlamydospores capable of infecting apple tree roots for a prolonged period, up to 10 years (Xiang et al., 2021a). The germination of *Fusarium* conidia is stimulated by root exudates, facilitating their penetration into the root system through wounds or natural openings, leading to rotting and necrosis. Additionally, *Fusarium* pathogens can also produce toxins that further contribute to the decline of the root tissues, thereby affecting the normal growth and development of apple and other plants (Dong et al., 2012; Crutcher et al., 2017; Manici et al., 2021). Therefore, *Fusarium* pathogens can be considered a crucial direct target for biological control of ARD. Presently, the primary commercially available approaches for ARD control involve fumigation and soil disinfestation using substances like dazomet, calcium cyanamide, or methyl bromide (Nicola et al., 2017; Winkelmann et al., 2019; Jiang et al., 2022). However, the environmental pollution and damage to soil microbial ecology associated with fumigation methods have raised concerns, leading to their gradual phasing out from agricultural practices (Raymaekers et al., 2020). In response to these challenges, the utilization of biological control agents (BCAs) is an increasingly popular and sustainable method in many countries for mitigating plant diseases due to its environmentally friendly and long-lasting efficacy (Massart et al., 2015). Consequently, the development of effective BCAs stands out as a crucial strategy for the management of ARD.

Currently, *Bacillus* spp. are gram-positive bacteria that have gained recognition and commonly employed as BCAs for their potential against a wide range of plant pathogens in agriculture (Chen et al., 2020; Etesami et al., 2023). They can establish mutualistic relationships with plants by colonizing their rhizosphere and root system, providing protection against pathogen attacks through mechanisms such as resource competition, the production of antimicrobial compounds and extracellular hydrolytic enzymes, and the induction of systemic resistance in plants (Jangir et al., 2018; Khan et al., 2018; Kashyap et al., 2022). Additionally, *Bacillus* species contribute to plant growth by facilitating nutrient solubilization, producing phytohormones, and enhancing stress tolerance (Sarangi and Ramakrishnan, 2023). Previous studies highlight the efficacy of *B. amyloliquefaciens* L3, isolated from watermelon rhizosphere, in inhibiting *F. oxysporum* f. sp. *Niveum* through the production of antifungal compounds (Wu et al., 2019). Similarly, the isolation of siderophore-producing *B. amyloliquefaciens* BM3 showed effective mitigation of arsenic contamination by over 70% and suppression of *Fusarium* wilt in brinjal plants (Pradhan et al., 2023). Through *in vitro* and pot experiments, Fan et al. (2023) found that a promising strain of *B. velezensis* called YN1910, which was obtained from disease-suppressive soils, not only significantly controlled banana *Fusarium* wilt (with an efficacy rate of 78.43%-81.76%), but also promoted the growth of banana plants. Excitingly, the latest research findings have reported a combination of *Bacillus* species that exhibit relative antagonism against common pathogens associated with ARD. The addition of a composite microbial culture containing 2.6×10^9^ CFU/g *Bacillus* spp. to the soil (along with a fertilizer carrier of cow manure and straw in a 3:1 ratio) significantly increased the biomass of ARD-affected *Malus hupehensis* Rehd. plants (Geng et al., 2022). However, the biocontrol resources of *Bacillus* species that have demonstrated biocontrol effects on the four common ARD-related *Fusarium* spp. are still scarce.

*B. amyloliquefaciens* has demonstrated potential and broad applicability in sustainable agriculture, but its effectiveness against the specific *Fusarium* that causes ARD has not been explored previously. In this study, a strain of *B. amyloliquefaciens* BA-4 that produces lytic enzymes and siderophore was isolated from the rhizosphere of healthy apple trees in orchard that were over 20 years old and needed replanting. The objectives of this study are as follows: (1) characterize the antagonistic activity of the bacterial isolate BA-4 against four ARD-related *Fusarium* pathogens; (2) evaluate the ability of strain BA-4 in promoting plant growth; (3) determine the biocontrol efficacy of BA-4 against ARD in both greenhouse and field settings; (4) assess the colonization ability of BA-4 under field condition. The identification of the antagonistic bacterium BA-4 from the apple rhizosphere holds promise for novel approaches in the ecological control of ARD.

## 2 Material and methods

### 2.1 Fungal pathogens, growth conditions, and inoculum preparation

Four ARD-related pathogens (*F. oxysporum*, *F. proliferatum*, *F. moniliforme*, and *F. solani*) used in this study were isolated from the rhizosphere and bulk soil of apple replanted orchards with ARD in the Taihang Mountain region. These pathogens have shown high pathogenicity towards *Malus robusta* Rehd. (MR) seedlings, which are predominantly used as rootstock in this apple producing area. They were preserved and provided by the Apple Disease and Biological Control Laboratory, College of the Plant Protection, Hebei Agricultural University. All cultures grown on potato dextrose agar (PDA; 200 g potato, 5 g beef extract, 20 g glucose, 20 g agar, pH 7.0) at 28°. To prepare the conidial suspension, the 8-mm diameter discs obtained from the edge of a 7-day old mycelium were then transferred to a 250 mL flask containing 100 mL of potato dextrose broth (PDB) and then incubated on a rotary shaker at 160 rpm for one week at room temperature. Afterward, the culture was filtered through a sterile Buchner funnel with 1.3 mm diameter holes. The resulting suspension was adjusted to a suitable concentration of 1×10^7^ spores/mL with sterile distilled water and a hemocytometer.

### 2.2 Isolation and screening of antagonistic bacteria

Soil samples collected from the rhizosphere of healthy apple trees with over 20 years of age in old orchards in the Taihang Mountain area of Hebei Province were subjected to serial dilution with sterile water to isolate antagonistic microorganisms. Briefly, 100 µL of each of the 10^−5^, 10^−6^, and 10^−7^ dilutions were spread onto Luria-Bertani (LB) agar plates (10 g tryptone, 5 g yeast extract, 10 g NaCl, 15 g agar in 1 L, pH 7.0), and incubated at 30° for 24 hours. Different single colonies were picked and streak purified on a new LB plate for culture. For the preparation of the bacterial cell suspension, the cultured bacterial broth was centrifuged at 10,000 rpm for 20 minutes. The pelleted cells were subsequently suspended in 10 mM PBS solution (130 mM NaCl, 7 mM Na_2_HPO_4_, and 3 mM NaH_2_PO_4_ in 1 L, pH 7.4), and the cell density was adjusted to 1 × 10^8^ CFU/mL. This standardized cell suspension served as the basis for subsequent experimental procedures. The antagonistic activity against fungal pathogens was assessed using the plate confrontation method (Li et al., 2021). A mycelial disc with a diameter of 8 millimeters for each fungal pathogen was placed at the center of potato dextrose agar (PDA) plates. Two sterilized filter papers (6 mm in diameter) were positioned equidistantly on either side of the fungal pathogen. Each filter paper was spotted with 10 μL of bacterial cell suspension. As a control, mycelial discs with a diameter of 8 mm for each fungal pathogen were cultured at the center of PDA plates, with sterile distilled water replacing bacterial cell suspension. The antagonistic ability was evaluated by the inhibition rate of biocontrol bacteria on fungal mycelium growth, and the inhibition rate (%) = (D1–D2)/D1×100%, where “D1” is the colony diameter of phytopathogens in the control and “D2” refers to the colony diameter in the treatment plate. The experiment was repeated three times.

### 2.3 Determination of antifungal spectrum of antagonistic strains

To assess the antifungal spectrum of BA-4, the plate confrontation method was employed to determine its antagonistic activity against five different apple pathogenic fungi: *Phytophthora cactorum*, *Valsa mali*, *Alternaria mali*, *Colletotrichum gloeosporioides*, and *Botryosphaeria dothidea*. These fungal strains were preserved in the Apple Disease and Biological Control Laboratory at Hebei Agricultural University. Each treatment was replicated three times. Measurements of the colony diameter of pathogens and the width of inhibition zones were taken, and inhibition rates were calculated using the formula described in Section 2.2.

### 2.4 Identification of strain BA-4

The bacterial strain BA-4 was cultured on liquid medium at a temperature of 28° for 24 hours. The colony morphology of BA-4 was observed using optical microscopy and Gram staining. To determine the physiological and biochemical characteristics of BA-4, it was grown for 24 hours and tested according to the methods described in Berger’s Manual for Systematic Identification (Krieg et al., 2010). Additionally, the molecular identification of the BA-4 isolate was conducted based on its *16S rRNA* and *gyrB* sequences. Polymerase chain reaction (PCR) was performed to amplify the *16S rRNA* and *gyrB* genes using the universal primers 27F (5’-AGAGTTTGATCCTGGCTCAG-3’)/1492R (5’-GGTTACCTTGTTACGACTT-3’) and UP1f (5’-GAAGTCATCATGACCGTTCTGCAYGCNGGNGGNAARTTYGA-3’)/UP2r (5’-AGCAGGGTACGGATGTGCGAGCCRTCNACRTCNGCRTCNGTCAT-3’), respectively. The PCR products were verified by agarose gel electrophoresis and then sent for sequencing at Huada Gene Co., Ltd. The obtained sequences were subjected to homology alignment analysis using the BLAST program on the NCBI website. A phylogenetic tree was constructed using the maximum likelihood (ML) method in MEGA 7.0 software, bootstrap analysis based on 1000 replicates.

### 2.5 Inhibitory effect of extracellular metabolites from BA-4 on Fusarium species

#### 2.5.1 Effect of strain BA-4 on *Fusarium* hyphae

In addition to using the plate confrontation assay mentioned section 2.2 to assess the inhibitory potential of strain BA-4 against *Fusarium* hyphal growth. Subsequently, mycelial samples from the periphery of the inhibition zone, where there was no contact with BV4 clones, were collected for observation under a light microscope. This allowed for a detailed examination of any morphological alterations induced by the extracellular metabolites produced by BA-4.

#### 2.5.2 Effect of cell-free culture filtrate on *Fusarium* spore germination

The conidia suspension of four *Fusarium* species (1 × 10^6^ spores/mL) was mixed thoroughly with an equal volume of undiluted or diluted cell-free culture filtrate of BA-4 in 96-well cell culture plates. Initially, 100 µL of the spore suspension was introduced into each well, and 100 µL of serially diluted cell-free culture filtrate was added to each well at concentrations of 50%, 20%, 10%, 5%, 2.5%, and 1.25% (v/v). Subsequently, 100 μL of the mixed solution was dropped on a concave slide. The concave slide was placed under 28°C for 24 hours and moisture-keeping conditions.

To evaluate the spore germination rate, the concave slide was maintained in a moist environment and incubated at 28° for 24 hours. Spores were considered germinated if the length of the germ tube equaled or exceeded twice the diameter of the spore. Sterile water instead of cell-free culture filtrate was used as the control. The percentage inhibition was determined using the formula: Inhibition rate = (the total number of spores-the number of germinated spores/the total number of spores) × 100%. To ensure statistical robustness, the total number of spores under investigation exceeded 200, with three replicates conducted for each treatment. To delve deeper into the effect of BA-4 extracellular metabolites on *Fusarium* spore germination, the morphological alterations of spores treated with 5% (v/v) cell-free culture filtrate were examined under a light microscope.

### 2.6 Plant growth-promoting traits of BA-4 *in vitro* assays

#### 2.6.1 Phosphate & potassium solubilization ability

The following assays were conducted with slight modifications (Cui et al., 2019) to assess specific traits: The phosphate-solubilizing capability of BA-4 was assessed using Pikovskaya (PVK) agar medium (10 g glucose, 5 g Ca_3_(PO_4_)_2_, 0.2 g NaCl, 0.5 g (NH_4_)2SO_4_, 0.1 g MgSO_4_·7H_2_O, 0.2 g KCl, 0.5 g yeast extract, 2 mg MnSO_4_, 2 mg FeSO_4_·7H_2_O, 25 mg Bromphenol blue, 15 g agar in 1L, pH 7.2). A bacterial cell suspension of BA-4 (10 μL) was inoculated onto PVK plates and incubated at 28°C for 7 days to evaluate its ability to utilize inorganic phosphate from Ca_3_(PO_4_)_2_ as the sole phosphate source. Similarly, the potassium-solubilizing capability of BA-4 was determined on potassium feldspar (PF) agar medium (10 g sucrose, 0.5 g MgSO_4_·7H_2_O, 0.2 g (NH_4_)_2_SO_4_, 0.1 g NaCl, 0.1 g CaCO_3_, 5.0 g Potassium feldspar, 25 mg Bromophenol blue, 15 g agar in 1 L, pH 7.2). The appearance of a clear zone around the bacterial colony after 7 days at 28°C were considered positive for its ability to solubilize Phosphate and potassium.

#### 2.6.2 Nitrogen fixation ability

Nitrogen fixation ability was evaluated by streaking BA-4 colonies on nitrogen-free Ashby medium (5 g glucose, 5 g mannitol, 0.1 g CaCl_2_·2H_2_O, 0.1 g MgSO_4_·7H_2_O, 5 mg Na_2_MoO_4_·2H_2_O, 0.9 g K_2_HPO_4_, 0.1 g KH_2_PO_4_, 0.01 g FeSO_4_·7H_2_O, 5 g CaCO_3_, 15 g agar in 1L, pH 7.2) and culturing them at 28°C for 7 days. The observation of clear zone around bacterial colonies was considered positive for its nitrogen fixation ability.

#### 2.6.3 Indole-3-acetic acid production

The production of indole-3-acetic acid (IAA) analogue by BA-4 was detected using Landy liquid medium (20 g glucose, 5 g glutamic acid,1 g yeast extract, 5.0g L-glutamic acid, 2mg L-phenylalanine, 1.0g L-tryptophan, 1 g K_2_HPO_4_, 0.5 g MgSO_4_·7H_2_O, 0.5 g KCl, 0.16 mg CuSO_4_·7H_2_O, 0.15 mg Fe_2_(SO_4_)_3_·7H_2_O and 4 mg MnSO_4_·4H_2_O in 1L, pH 7.2). BA-4 was cultured in Landy liquid medium with or without L-tryptophan for 3 days at 28°C, 160 rpm on a rotatory shaker. Subsequently, the culture supernatant was mixed with Salkowski’s reagent (150 mL concentrated H_2_SO_4_, 7.5 mL 0.5M FeCl_3_·6H_2_O and 250 mL ddH_2_O) in a 1:2 ratio, and the optical absorbance of the resulting mixture was measured at 530nm. The color intensity was directly proportional to the IAA concentration, which was quantified by referencing a standard curve.

#### 2.6.4 Test of siderophore production

The production of siderophores by BA-4 was assessed using Chrome chrome azurol sulphonate (CAS) agar medium (60.5 mg CAS, 72.9 mg hexadecy-ltrimethyl-ammonium bromide, 2.65 mg FeCl_3_·6H_2_O, 295.2 mg NaH_2_PO_4_·2H_2_O, 1213 mg Na**_2_** HPO_4_·12H_2_O, 125 mg NH_4_Cl, 37.5 mg KH_2_PO_4_, 62.5 mg NaCl, 15 **g** agar in 1L, pH 7.2). A bacterial cell suspension of BA-4 (10 μL) was inoculated onto CAS plates and incubated at 28°C for 2 days. The formation of an orange zone around the colony indicated the presence of siderophore production.

### 2.7 Detection of extracellular enzymes of BA-4 *in vitro* assays

The enzymatic activities of BA-4, focusing on protease and cellulase, were investigated using skimmed milk agar medium (5 **g** skimmed milk powder, 10g glucose, 1g yeast extract, 1g K_2_HPO_4_, 0.5 **g** KH_2_PO_4_, 0.5 **g** MgSO_4_·7H_2_O, 15 **g** agar in 1L, pH 7.2) and carboxymethyl cellulose (CMC) agar medium (15 **g** CMC-Na, 2.5 **g** Na_2_HPO_4_, 2 **g** KH_2_PO_4_, 0.5 **g** MgSO_4_·7H_2_O, 2 **g** peptone 15 **g** agar in 1L, pH 7.2), respectively. For cellulolytic activity assessment, a 10 μL suspension of strain BA-4 was blotted and then cultured on CMC media plates at 28°C for 3 days. After incubation, the ability to degrade CMC of BA-4 were examined by flooding the plates with a 0.5% Congo red solution. Following a 10-minute immersion period, the plates were washed with 1M NaCl. The presence of haloes surrounding the colonies indicated cellulase production. Protease production was measured in skimmed milk medium, and positive activity was determined by the formation of a clear halo around each colony.

### 2.8 Evaluation of plant growth-promoting activity of strain BA-4

#### 2.8.1 Growth-promoting ability of strain BA-4 on mung bean seedlings

For the mung bean seedling experiment, mung bean seeds with consistent growth conditions of root length and bud length were selected as experimental materials. These seeds were then planted in plastic pots filled with sterilized seedling substrate. The plants were cultivated in a greenhouse under standard conditions, maintaining a temperature of 28°C, humidity at 90%, and a light-dark (L: D) ratio of 16:8. Upon reaching the 3-leaf stage, the mung bean plants underwent irrigation with various treatments. The treatments encompassed irrigating the seedlings with sterile water as a negative control and applying different concentrations (2.0 × 10^7^ CFU/mL, 1.0 × 10^8^ CFU/mL, and 5.0 × 10^8^ CFU/mL) of the BA-4 bacterial suspension as treatment groups. Each plant received an inoculation of 10 mL/50g of soil by root- irrigation, and this procedure was repeated twice with a one-week interval between repetitions. There were 12 bean plants in each treatment, and the experiment was replicated three times. On the 14th day post-treatment, various growth indices such as plant height, fresh weight, dry weight, primary root length, lateral root number and root weight were measured. The concentration of the BA-4 suspension that exhibited optimal growth promotion was selected for the subsequent MR seedlings experiment.

#### 2.8.2 Growth-promoting ability of strain BA-4 on *Malus robusta* Rehd. seedlings

For the MR seedling experiment, surface-sterilized seeds were vernalized at 4°C for approximately 45 days before being planted in flowerpots filled with sterilized seedling substrate. Upon reaching the stage of four true leaves, uniformly sized and healthy plants were chosen for the treatment. Subsequently, the selected plants underwent irrigation with sterile water as a negative control, and an optimal bacterial suspension of strain BA-4 at a concentration of 1.0 × 10^8^ CFU/mL. The inoculation amount for each plant was 10 mL/50g soil by root- irrigation, which was repeated twice with a two-week interval. Temperatures of the greenhouse were maintained at 25° (day) and 20° (night), humidity at 80%, and a light-dark (L: D) ratio of 16:8. The plants were consistently watered based on their requirements. In each treatment, there were 9 MR plants, and the experiment was replicated three times. On the 30th day post-treatment, various growth parameters, including plant height, fresh weight, primary root length, lateral root number and dry weight, were measured. The growth of the seedlings was closely monitored, and any variations in growth rate or other parameters were systematically recorded and compared to untreated seeds during test.

### 2.9 Evaluation of control effect of BA-4 against apple replant disease

#### 2.9.1 Control effect of BA-4 against specifical *Fusarium*-caused disease in the greenhouse

The pot experiment was performed at the Apple Pest and Disease Control Experiment Center of the Plant Protection College of Hebei Agricultural University (Lon: 115.447448 Lat: 38.827133). MR seedlings (4–5-leaf-stage) were transplanted into a pot (10 cm × 12 cm) containing 200 g of autoclaved soil, 1 plant/pot. For protective test, seedlings were inoculated with 40 mL BA-4 suspension (1.0 × 10^8^ CFU/mL) by root-irrigation; sterile water as negative control. After 1 day, *Fusarium* spores were inoculated on the roots by irrigating. For therapeutic test, seedlings were inoculated first with the *Fusarium* spores. On the third day following the inoculation of pathogenic spores, the BA-4 suspension was inoculated in the absence of ARD symptoms, sterile water as negative control. Root irrigation with BA-4 suspension was repeated at intervals of 2 weeks. There were 12 MR seedlings in each treatment, and the experiment was repeated three times. Disease severity index was assessed over a 5-week period, starting 1 week after inoculation. The revised disease severity level standard for ARD is outlined as follows (Cheffi Azabou et al., 2020): 0 = a healthy plant or a plant without symptoms; 1 = 1–33% of plant tissue affected by chlorosis, leaf and shoot necrosis, or defoliation; 3 = 34–66% affected tissue;5 = 67–100% affected tissue; and 7 = a dead plant. The disease index and relative control effect were calculated. The incidence of new plants was monitored, and the duration of each treatment for typical ARD was analyzed. The disease index is computed using the formula: Disease index = ∑(Number of diseased seedling × Number of Grade)/(Total number of seedling × Highest number of Grade). Biocontrol efficacy is determined by the formula: Biocontrol efficacy (%) = (Disease index of control − Disease index of treatment)/Disease index of control × 100. The whole experiment was set according to a completely randomized block design, and the experiment was repeated independently in April of the following year.

#### 2.9.2 Control effect of BA-4 against apple replant disease in the field

To assess the efficacy of *B. amyloliquefaciens* strain BA-4 in managing ARD, the replant soil samples were gathered from a 30-year-old orchard in Baoding, Hebei (Lon: 114.977931, Lat: 38.785267) known to be infected with *Fusarium* spp. The region has a monsoon continental climate, with average temperatures of 10.4°C and annual precipitation of 594.3 mm respectively. These replant soil samples, collected within an 80 cm radius around the trunk and at a depth of 20-40 cm below the soil surface, were then transported to fill a deep ditch in the orchard of Hebei Agricultural University in April 2021. Physicochemical properties of the tested soil indicated a pH level of approximately 8, with soil nitrogen concentration at 1.13g/kg, organic matter content at 12.52 g/kg, available phosphorus at 108.12 mg/kg, available potassium at 264.73 mg/kg, available iron at 11.36 mg/kg, and available zinc at 4.02 mg/kg, respectively. The purpose of backfilling the replanted soil was to replicate the field environment for studying the ability of strain BA-4 to control ARD. For field experiments, MR seedlings with comparable growth potential, reaching the stage of four true leaves, were selected as plant materials. The experimental treatments included the replant soil irrigated with the suspension of strain BA-4 and the replant soil irrigated with equal volume of sterile water as a control; the sterilized replant soil (fumigated with methyl bromide) irrigated with the suspension of strain BA-4 and the sterilized soil irrigated with equal volume of sterile water as a control. Each treatment involved 12 MR seedlings, with each plant in the BA-4 treatment group receiving irrigation of 300 mL suspension with a concentration of 1 × 10^8^ CFU/mL or sterile water. Root irrigation was repeated twice at monthly intervals, and subsequent watering was based on the plants’ growth and disease development requirements. After a three-month period following root irrigation with the BA-4 suspension, disease severity was assessed by measuring mortality, plant height, leaf numbers, ground diameter, root length, and chlorophyll content.

### 2.10 Evaluation of colonization ability of antibiotic labeled BA-4 strain

To evaluate the root colonization ability of *B. amyloliquefaciens* BA-4, an antibiotic labeling (AL) and root colonization assay was carried out. Initially, a rifampin-resistant labeling strain, BA-4AL, was generated by exposing strain BA-4 to increasing concentrations of rifampin (1, 5, 10, 20, 40, 60, 80, 100, 150, and 200 µg mL-1) in LB medium, following the methodology outlined by (Duan et al. (2022a). Subsequently, BA-4AL strains underwent five consecutive subcultures on LB agar plates containing 200 µg/mL of rifampicin. The fifth-generation strains were employed to evaluate the stability of antibiotic labeling and its impact on BA-4. This assessment included a comparison of morphological differences, drug resistance, and antagonistic capabilities between the fifth-generation resistant labeling strain BA-4AL and BA-4.

The colonization and dynamics of BA-4AL in the soil surrounding the roots of MR seedlings were evaluated using the root irrigation method. In March 2022, healthy and uniform 6-leaf MR seedlings were transplanted into the experimental garden at Hebei Agricultural University. Each treatment was replicated with 12 seedlings. Two types of plots were utilized for transplanting MR seedlings: a normal replant soil plot, where apple trees with MR as rootstock had been planted for 16 years, and another sterilized replant soil plot, where the soil was chemically fumigated with methyl bromide. One month after planting, seedlings were subjected to root irrigation. Bacterial suspensions of BA-4AL, prepared as described previously, included the addition of rifampicin at 200 µg/mL to the medium. Specifically, 300 mL of BA-4AL suspension (2 × 10^8^ CFU/mL) was applied to the root soil of each plant. Control treatments mimicked the process, with the only variation being that roots were irrigated with 300 mL of sterile distilled water instead of the BA-4AL suspension. Soil samples were collected from each seedling rhizosphere in both plots at depths of 0-10 cm and 10-20 cm on days 1, 2, 3, 5, 10, 15, 20, 30, 60, and 90 post-root inoculation. The soil suspensions were prepared, diluted with PBS buffer, and subjected to serial dilutions plated onto LB agar containing 200 µg/mL rifampicin for 24 hours at 37°C. The number of labeled BA-4AL colonies was recorded, and bacterial populations were expressed as CFU/g of dried soil. Finally, strain BA-4AL was re-isolated and confirmed through physiological and biochemical characteristics, as well as a plate confrontation assay.

### 2.11 Statistical analysis

The statistical analysis of the data was performed using SPSS 17.0 software (SPSS Inc., Chicago, IL, USA). Mean values were compared using Duncan’s Multiple Range Test at *p* < 0.05. Figures were generated using Microsoft Excel 2013 (Microsoft Corporation, Redmond, Washington, USA) and GraphPad Prism 7.0 (GraphPad software, Inc., San Diego, California, USA).

## 3 Results

### 3.1 Isolation and evaluation of antagonistic bacteria

**Figure 1:**
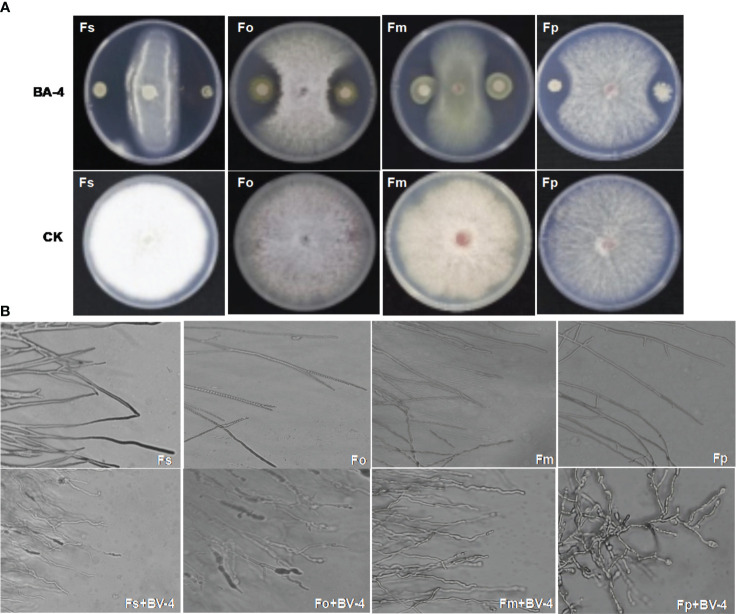
Antagonistic of *Bacillus amyloliquefaciens* BA-4 against four *Fusarium* pathogens. **(A)** Plate confrontation assay between BA-4 and pathogenic fungi *in vitro* (Fo: *Fusarium oxysporum*, Fm: *Fusarium moniliforme*, Fp: *Fusarium proliferatum*, and Fs: *Fusarium solani*). **(B)** The effect of strain BA-4 on the mycelia morphology of four *Fusarium* pathogens. the upper part was the normal mycelium, while the lower part was the mycelium confronted with BA-4 colonies.

Bacterial strains isolated from apple rhizosphere soil from diverse orchards displayed varying degrees of biocontrol potential. A total of 159 bacterial isolates were obtained and screened in the dual culture test for potential antagonistic activity against ARD-related *Fusarium* pathogens, including *Fusarium oxysporum*, *F. proliferatum*, *F. solani*, *F. moniliforme*. Among these isolates, the BA-4 strain isolated from the rhizosphere soil of healthy apple trees within a 15-year-old orchard in Baoding City, Hebei Province, which displayed broad-spectrum antagonistic activity against the four *Fusarium* pathogens and surpassed other isolates in inhibition efficiency (**Figure 1A**). In addition, strain BA-4 exhibited notable inhibitory effects on the five other important apple pathogens *Phytophthora cactorum*, *Valsa mali*, *Alternaria mali*, *Colletotrichum gloeosporioides*, *and Botryosphaeria dothidea*, with respective inhibition of 74.8, 70.5, 65.8, 86.9 and 81.2% (**Table 1**). These results highlight the wide-ranging antagonistic capabilities of BA-4 strain *in vitro*. Consequently, strain BA-4 was chosen for subsequent assessments of its potential in controlling ARD.

**Table 1:** Inhibitory effects of strain BA-4 on the mycelial growth of phytopathogenic Fungi.

Phytopathogenic fungi	Inhibition rate (%)
*Fusarium oxysporum*	54.7 ± 4.4
*F. proliferatum*	50.2 ± 5.1
*F. moniliforme*	63.1 ± 2.8
*F. solani*	74.7 ± 4.2
*Alternaria mali*	74.8 ± 4.5
*Botryosphaeria dothidea*	70.5 ± 3.8
*Colletotrichum gloeosporioides*	65.8 ± 6.1
*Phytophthora cactorum*	86.9 ± 3.9
*Valsa mali*	81.2 ± 4.7

Values are means ± standard error of the mean (n = 6).

### 3.2 Physiological and biochemical characteristics of strain BA-4

**Figure 2:**
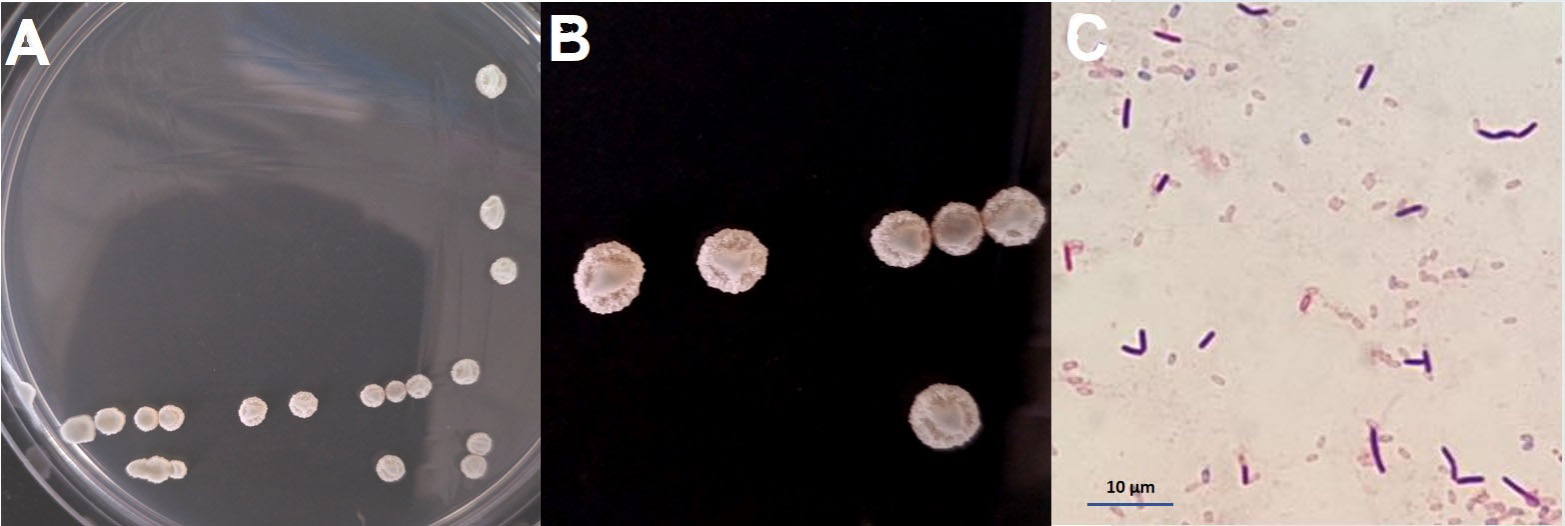
Morphological characteristics of *Bacillus amyloliquefaciens* BA-4. **(A, B)** Colony morphology of strain BA-4 on LB solid medium; **(C)** Gram-positive staining of BA-4, scale bar 10.0 µm.

Following 24 hours of cultivation on LB agar at 30°, BA-4 single colony appeared a nearly circular shape with a pale yellow color, raised center, wrinkled and rough surfaces, presenting an opaque appearance (**Figures 2A, B**). The cells displayed a straight, rod-shaped morphology, and strain BA-4 was identified as a gram-positive strain (**Figure 2C**).

The outcomes of biochemical and physiological examinations for strain BA-4 are outlined in **Table 2**. HSB-2 demonstrated proficiency in hydrolyzing starch, reducing nitrate, and utilizing mannitol, fructose, glucose, sucrose, or maltose as a carbon source. Positive results were observed for gelatin liquefaction, Voges-Proskauer reaction, and citrate utilization, but Methyl red test yielded negative outcomes. In accordance with Bergey’s Manual of Systematic Bacteriology (2nd edition) and the Common Bacterial Identification Manual, these characteristics of BA-4 closely resembled those identified in *Bacillus* species (**Table 2**).

**Table 2:** Physiological and biochemical characterization of strain BA-4.

Test	Reaction^a^	Test	Reaction^a^
Gram stain	+	pH 5	+
Voges-Proskauer (VP) test	+	pH 7	+
Citrate	+	pH 8	+
Methyl red test	−	pH 9	−
V-general test	+	20 °C	+
Nitrate reductase	+	30 °C	+
Starch hydrolysis	+	40 °C	+
Gelatin liquefaction	+	60 °C	−
Glucose fermentation	+	2% NaCl	+
Fructose fermentation	+	5% NaCl	+
Mannitol fermentation	+	7% NaCl	+
Sucrose fermentation	+	10% NaCl	−
Maltose fermentation	+		

a + and − represent positive and negative reactions, respectively. Each test was conducted in three independent experiments.

### 3.3 Molecular identification of the bacterial strain BA-4

**Figure 3:**
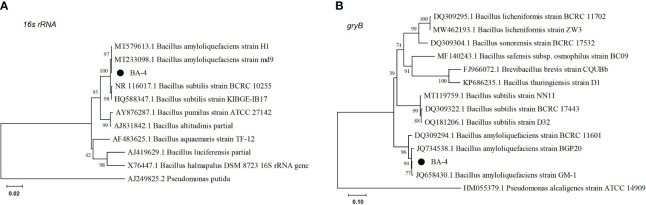
Phylogenetic trees were constructed based on *16S rRNA* **(A)** and *gyrB* **(B)** gene sequences. Phylogenetic trees were constructed by the maximum likelihood method of MEGA 7.0 with bootstrap values based on 1000 replications.

Sequencing results of the *16S rRNA* and *gyrB* genes of the strain BA-4 were subjected to BLAST analysis against the NCBI nucleotide database. The results showed a sequence similarity of 99% with *B. amyloliquefaciens*. A phylogenetic tree was created using partial *16S rDNA*, and *gyrB* sequences, in conjunction with closely related genetic sequences. The phylogenetic analysis demonstrated that Strain BA-4 formed a branch within *B. amyloliquefaciens* strains (**Figure 3**), indicating its close relation to *B. amyloliquefaciens*. Consequently, Strain BA-4 was conclusively identified as *B. amyloliquefaciens*.

### 3.4 Effects of strain BA-4 on mycelium and conidia of *Fusarium* species

**Figure 4:**
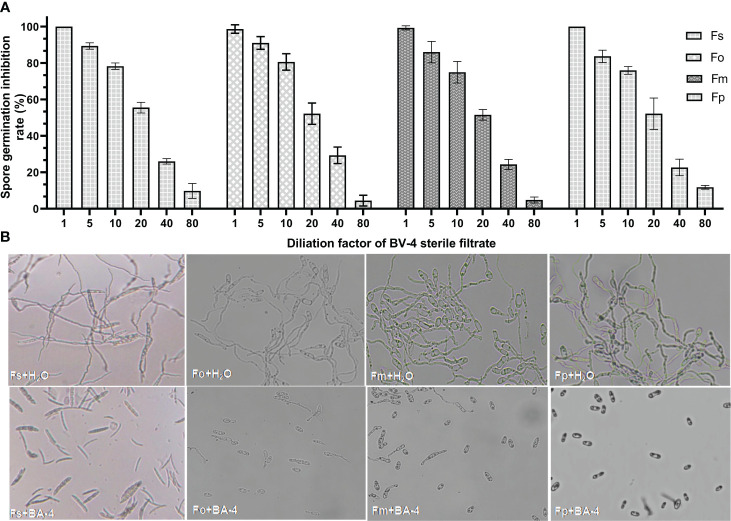
The effect of strain BA-4 on the spore germination of four *Fusarium* pathogens. **(A)** Spore germination inhibition rate after treatment with different concentrations of BA-4 cell-free culture filtrate. **(B)** Effect of cell-free culture filtrate of BA-4 on *Fusarium* spore morphology. The upper part was normal spores, while the lower part was spores treated with 5% BA-4 cell-free culture filtrate.

The strain BA-4 exhibited strong inhibitory effects on the mycelial growth and spore germination of *Fusarium* pathogens. In the control group of the confrontation experiment, the pathogenic hyphae exhibited uniform and slender characteristics, with minimal branching, well-developed spores, and an intact structure (**Figures 1B**, **4B**). Conversely, in the treatment group, all *Fusarium* pathogens along the BA-4 colony border displayed inhibitory areas (**Figure 1A**). The hyphae on the confrontation side exhibited irregular mesh distribution, uneven thickness, contraction, thinning, fracture, and leakage of cell content (**Figure 1B**). Following treatment with cell-free culture filtrate of strain BA-4, the spore germination of all four *Fusarium* pathogens was mostly inhibited (**Figure 4A**). Even when the BA-4 fermentation broth was diluted by 20 times, the spore germination rate remained more than 50% lower than that in the control group (**Figure 4A**). Microscopic examination of spore germination indicated that, after treatment with BA-4 sterile filtrate, even germinating spores had shorter germ tube segments compared to the control (**Figure 4B**). These observations suggest that metabolites produced during the fermentation of BA-4 might have a profound impact on the spore germination and normal growth of *Fusarium* pathogens. Overall, these preliminary findings underscore the broad-spectrum inhibitory effects of the BA-4 strain against the *Fusarium* pathogens associated with ARD, highlighting its potential as a versatile agricultural biocontrol agent.

### 3.5 Characterization of potential plant-beneficial traits of strain BA-4

**Figure 5:**
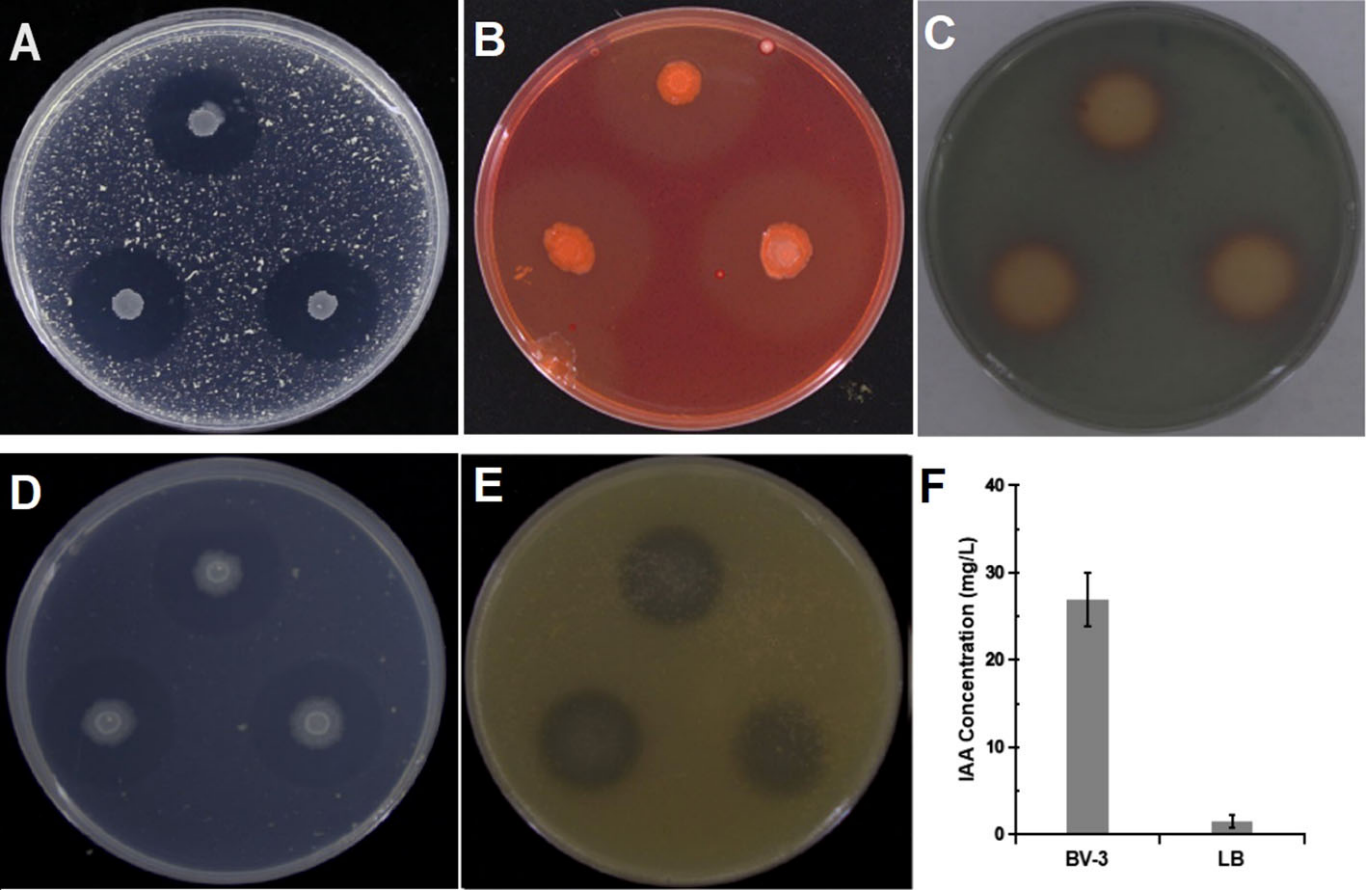
Detection of cell wall-degrading enzymes and plant growth-promoting traits. **(A)** protease activity; **(B)** cellulase activity; **(C)** siderophore production; **(D)** phosphate solubilization; **(E)** potassium solubilization; **(F)** indole-3-acetic acid **(IAA)** production.

Several traits of BA-4 strain involved in plant growth promotion and disease prevention were tested *in vitro*. The observation of clear zones formed around BA-4 strain colonies grown on specific agar medium indicated its ability to dissolve potassium, solubilize phosphate, and produce cellulase and protease. Siderophore production was confirmed by an orange-yellow area around BA-4 colonies on CAS agar medium (**Figures 5A-E**). However, nitrogen-fixing capabilities were not observed on nitrogen-free medium. Additionally, BA-4 exhibited a positive biosynthetic capability for IAA-like molecules using L-tryptophan as a crucial precursor, and the production of IAA reached 47.8 mg/L (**Figure 5F**).

### 3.6 BA-4 suspension promotes growth of bean and *Malus robusta* Rehd. seedlings

The agronomic traits (including fresh weight, dry weight, number of fibrous roots, root length, and root weight) of bean seedlings treated with BA-4 suspension at different concentrations were significantly better than those of water control (**Table 3**). Overall, the best growth-promoting effect of the biocontrol strain BA-4 was observed at a concentration of 1 ×10^8^ CFU/mL, and higher concentration of BA-4 may pose a risk of harmful plant growth (**Table 3**). Quantitative results showed that the plant height, fresh weight, dry weight, root length, fibrous root number, root length, and root weight of bean seedlings treated with BA-4 suspension (1×10^8^ CFU/mL) increased by 50.29%, 78.77%, 212.64%, 23.88%, 143.83%, and 153.34%, respectively, than water treatment (**Table 3**). In particular, the root length and root weight of bean seedlings increased by more than 100%, indicating that strain BA-4 has a more significant positive effect on root development (**Table 3**). Therefore, subsequent growth promoting experiment on MR seedlings were conducted at this concentration of 1 ×10^8^ CFU/mL.

**Table 3:** Effect of BA-4 Suspension on the growth of Mung Bean Seedlings.

Treatments	Concentration (CFU/mL)	Plant height (mm)	Fresh weight (mg)	Dry weigh (mg)	Number of fibrous roots	Root length (mm)	Root weight (mg)
**BA-4**	2×10^7^	116.89 ± 12.04a	632.96± 78.72a	68.11 ± 10.37b	12.72 ± 1.83a	34.63± 5.16b	56.35 ± 23.32b
	1×10^8^	169.17 ± 15.96b	917.13 ± 94.98b	195.96 ± 50.16c	21.89 ± 3.04c	56.74 ± 7.22c	65.31 ± 11.16c
	5×10^8^	156.89 ± 7.30b	681.89 ± 60.85b	44.96 ± 7.95a	15.95 ± 3.37a	19.60 ± 1.78a	29.76 ± 8.71a
**CK**	sterile water	112.56 ± 15.42a	513.01 ± 55.08a	62.68 ± 10.45b	17.67 ± 1.45ab	23.27 ± 2.24a	25.78 ± 2.86a

Different lowercase letters above the columns indicate a significant difference at p < 0.05. Numerical values were mean ± SD of triplicates.

**Figure 6:**
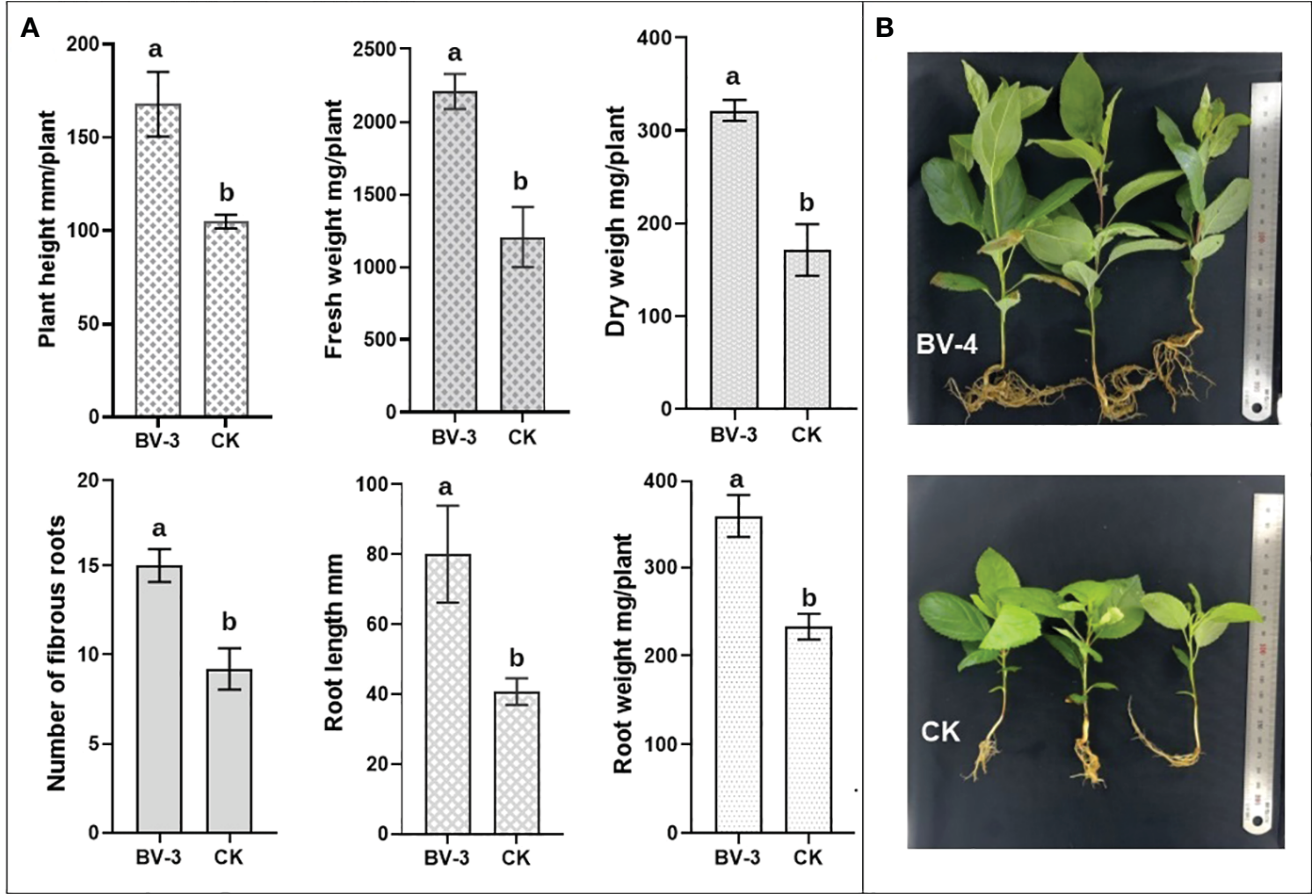
Effect of BA-4 Suspension on the growth of *Malus robusta* Rehd. seedling. **(A)** Growth-related indicators, including plant height, fresh weight, dry weight, number of fibrous roots, root length, and fresh weight. Different lowercase letters indicate a significant difference at *p* < 0.05. The values were mean ± SD of triplicates. **(B)** Observation of seedling growth under BA-4 suspension treatment and non-treatment conditions.

The results of the potted experiment indicate that the BA-4 suspension significantly enhanced both the aboveground growth of MR seedlings. After 30 days of BA-4 treatment, there was a substantial increase in plant height and weight, with increments exceeding 60% and 80%, respectively (**Figure 6A**). Furthermore, there was a positive effect of BA-4 suspension on the root development of MR seedlings. Compared to the water control, the number of fibrous roots, primary root length and dry weight increased by 63.14%, 96.70% and 53.70% in the treatment group, respectively (**Figure 6A**). Furthermore, the MR seedlings treated with strain BA-4 exhibited robust characteristics, featuring enlarged and dark green leaves, and well-developed roots (**Figure 6B**). These observations suggest that the application of the BA-4 strain is conducive to the overall growth and root development of MR seedlings.

### 3.7 Biocontrol of strain BA-4 on apple replant disease under greenhouse conditions

**Figure 7:**
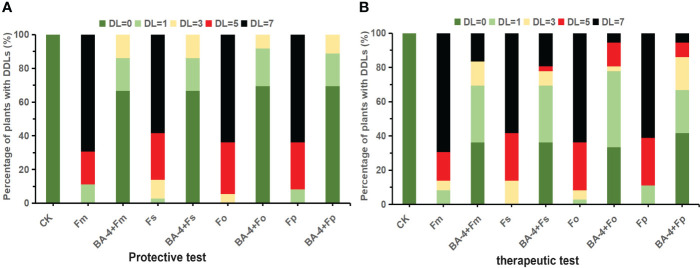
Effect of BA-4 on disease severity levels (DDLs) of replanted *Malus robusta* Rehd. (MR) seedling with or without BA-4 treatment. **(A)** Distribution of DDLs of MR seedlings in protective experiments; **(B)** Distribution of DDLs of MR seedlings in therapeutic experiments. CK: not inoculated with *Fusarium* spores.

Initially, pot experiments were conducted to explore the potential protective effects of the BA-4 strain suspension in suppressing *Fusarium*-related ARD. One week post-inoculation with *Fusarium* spore suspension, the positive control MR seedlings manifested classic wilt symptoms. In the subsequent 5 weeks, the incidence of *Fusarium* wilt escalated rapidly, reaching full disease manifestation, with the majority of plants reaching disease severity levels of 4 by the end. Similar ARD symptoms were noted in plants treated with BA-4, but at a markedly reduced intensity. These plants predominantly displayed milder symptoms, concentrated at lower disease severity levels (**Figure 7**). The disease severity index in *Fusarium* inoculation controls exhibited a gradual increase throughout the experimental period, ultimately reaching 41.61, 40.83, 43.16, and 41.61, respectively. In contrast, plants treated with BA-4 showed a reduction in disease progression, with the protection efficacy of strain BA-4 against *Fusarium*-related ARD approaching 90% (**Table 4**).

**Table 4:** Control effect of BA-4 on *Fusarium-*related apple replant disease in the greenhouse.

		Treatments							
		Fm	BA-4+Fm	Fs	BA-4+Fs	Fo	BA-4+Fo	Fp	BA-4+Fp
**2022**	**Disease index**	41.61 ± 3.37	4.27 ± 0.91	40.83 ± 3.11	4.27 ± 0.90	43.16 ± 0.77	3.31 ± 0.25	41.61 ± 1.03	3.69 ± 0.64
	**Protective** **efficacy (%)**	–	89.46 ± 2.85	–	89.62 ± 1.70	–	92.32 ± 0.72	–	91.12 ± 1.69
	**Disease index**	41.61 ± 1.03	13.41 ± 1.16	41.22 ± 1.29	14.58 ± 2.72	42.38 ± 2.59	11.27 ± 1.29	40.44 ± 2.07	9.52 ± 0.91
	**Therapeutic** **efficacy (%)**	–	67.81 ± 2.41	–	64.71 ± 6.37	–	73.09 ± 4.28	–	76.47 ± 1.46
**2023**	**Disease** **index**	38.11 ± 4.76	4.66 ± 0.38	35.02 ± 6.74	6.23 ± 0.94	42.78 ± 7.91	4.67 ± 1.03	44.33 ± 5.95	3.89 ± 0.78
	**Protective** **efficacy (%)**	–	87.75 ± 5.74	–	82.22 ± 7.03	–	89.09 ± 8.75	–	91.12 ± 5.97
	**Disease index**	44.33 ± 5.97	14.78 ± 1.02	39.67 ± 2.17	18.67 ± 2.89	42.78 ± 2.74	20.22 ± 1.95	37.34 ± 1.85	11.67 ± 0.65
	**Therapeutic** **efficacy (%)**	–	66.67 ± 6.02	–	52.94 ± 3.16	–	52.72 ± 3.06	–	68.75 ± 1.23

Numerical values were mean ± SD of triplicates.

Additionally, in the pot experiment assessing the therapeutic effect of the strain BA-4 on ARD, comparable outcomes were observed. There was a noteworthy decrease in disease severity among BA-4 treated plants, with a therapeutic efficacy ranging from 64.71% to 76.47% (**Table 4**). Subsequent repeated pot experiments in the second year substantiated the protective influence of BA-4 against *Fusarium*-related ARD. The control effects for both protective and therapeutic tests at 30th day exceeded 80% and 50%, respectively. Furthermore, plants treated with distilled water remained healthy, and at the conclusion of the experiment, pathogenic *Fusarium* was successfully re-isolated from the inoculated plants, confirming that the symptoms were induced by artificial infection with *Fusarium* pathogens. Overall, the results demonstrated that root irrigation with BA-4 suspension provided protection to apple plants against attacks from four *Fusarium* species throughout the testing period.

### 3.8 Biocontrol of strain BA-4 on apple replant disease under field conditions

**Figure 8:**
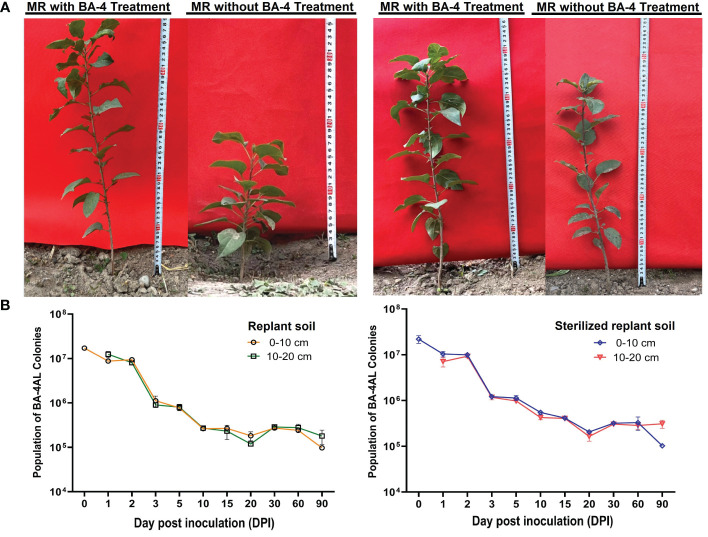
Effect of the BA-4 on the growth of *Malus robusta* Rehd. seedlings **(A)** and its colonization in the apple rhizosphere **(B)**. Replant soil: Soil from a 30-year-old orchard; Sterilized replant soil: The replanted soil is treated with methyl bromide fumigation; MR: *Malus robusta* Rehd. seedling.

Due to the absence of notable acute wilting symptoms, the field experiment initially focused on investigating cumulative mortality. Throughout the test period, plants treated with BA-4 exhibited a 33.34% reduction in mortality rate. Moreover, biomass measurements conducted after treatment with BV-4 for 3 months revealed a significant enhancement in plant height, leaf numbers and chlorophyll content in apple plants grown in normal replant soil, with increases of 53.98%, 15.45% and 28.48%, respectively (**Figure 8A** and **Table 5**). When MR seedlings were planted in sterilized replanted soil, it was observed that there was no seedling mortality in either water or BA-4 treatment at the conclusion of the test. However, BA-4 treatment resulted in substantial increases in plant height compared to sterile water controls (**Figure 8A** and **Table 5**), indicating that BA-4 has a certain growth-promoting effect on apple plants. In summary, these findings suggest that BA-4 treatment effectively mitigated the symptoms and severity of ARD under field conditions, highlighting its considerable potential for biological control.

**Table 5:** Effect of BA-4 on biomass and mortality rate of replanted *Malus robusta* Rehd. seedling in the field.

Treatments		Plant height (mm/plant)	Leaf Numbers	Stem diameter (mm)	Chlorophyll content (SPAD)	mortality rate (%)
**Replant soil**	**BA-4**	46.65 ± 4.53b	27.45 ± 6.51a	4.34 ± 0.63a	59.51 ± 4.07c	19.44 ± 3.70b
	**CK**	21.47 ± 3.74a	23.21 ± 4.67a	3.99 ± 0.40a	42.56 ± 1.27a	52.78 ± 9.26c
**Sterilized** **replant soil**	**BA-4**	59.43 ± 9.34c	38.18 ± 3.58b	5.22 ± 0.67b	55.10 ± 3.04bc	2.78 ± 3.70a
	**CK**	47.44 ± 5.30b	25.12 ± 2.96a	4.67 ± 0.72a	50.35 ± 5.68b	5.56 ± 3.70a

Different lowercase letters above the columns indicate a significant difference at p < 0.05. Numerical values were mean ± SD of triplicates. Replant soil: Soil from a 30-year-old orchard; Sterilized replant soil: The replanted soil is treated with methyl bromide fumigation; BA-4: 300 mL suspension containing a concentration of 1 × 10^8^ CFU/mL applied to each plant through root irrigation; CK: Irrigation with sterile water.

### 3.9 Colonization of strain BA-4 in apple rhizosphere soil

After five generations of continuous cultivation in a rifampicin-containing medium, the constructed rifampicin-resistant mutant strain BA-4AL exhibited no observable differences in colony morphology, antimicrobial activity, and growth curve when compared to the rifampicin-resistant mutant strain and the wild-type isolate. This indicates good genetic stability of its resistance, making it suitable for studying the colonization abilities of BA-4 in apple rhizosphere soil. BA-4AL-labeled strains were inoculated into apple trees grew in both normal and sterilized replant soil. From 0-90 d after inoculation, labeled strains were consistently and successfully re-isolated from the rhizosphere soil of MR seedlings. Analysis of the initial quantity of labeled strains in apple rhizosphere soil in normal replant soil revealed at 1.71×10^7^ CFU/g (1th day) and 1.23 ×10^7^ CFU/g (2th day) at depths of 0-10 cm and 10-20 cm, respectively. The number of BA-4AL strains rapidly decreased in the following 5 days after inoculation, stabilizing between 10^5^~10^6^ CFU/g at depths of 0-10 cm and 10-20 cm. From the 10th to the 90th day, the quantity of BA-4AL in replant soil slightly fluctuated, maintaining at about 10^5^ CFU/g (**Figure 8B**). In sterilized replant soil, the initial population of labeled strains in apple rhizosphere soil reached 2.18 ×10^7^ CFU/g (1th day) and 7.07 ×10^6^ CFU/g (2th day) at depths of 0-10 cm and 10-20 cm, respectively. On the 90th day, the population of BA-4AL dropped and remained at about 10^5^ CFU/g (**Figure 8B**). Inspiringly, the survival of BA-4AL was not significantly difference between normal and sterilized replant soils. In comparison, at a depth of 10-20 cm, the quantity of BA-4AL labeled strains still accounts for approximately 1% to 2% of the initial quantity, much higher than the survival rate around 0.5% at 0-10 cm depth in both soils. These results indicate that BA-4AL has strong colonization abilities in the apple rhizosphere soil and tends to colonize in the 10-20 cm soil layer.

## 4 Discussion

Due to its broad microbicidal spectrum, safe application, and environmentally sustainable attributes, BCAs have emerged as a compelling substitute for chemical fungicides in recent agricultural practices (Manzar et al., 2022). BCAs have been widely employed to manage fungal diseases affecting both aboveground and underground parts of crops. It is widely acknowledged that bacterial genera such as *Streptomyces*, *Pseudomonas*, and *Bacillus* stand out as pivotal contributors to the production of active antimicrobial substances in this context (Vurukonda et al., 2018; Chen et al., 2020; Höfte, 2021). *Bacillus* species, in particular, have garnered significant research attention for advancing future biological control strategies (Shafi et al., 2017; Etesami et al., 2023; Poulaki and Tjamos, 2023). Currently, various antagonistic *Bacillus* species have been isolated and screened from soil, plants, and other environments, including *B. amyloliquefaciens* (Zheng et al., 2018; Wu et al., 2019; Jia et al., 2023; Marković et al., 2023), *B. velezensis* (Reyes-Estebanez et al., 2020; Chen et al., 2022; Yan et al., 2022), *B. subtilis* (Bais et al., 2004; Ding et al., 2017; Qiao et al., 2023), among others. Previous investigations highlight the inhibitory and anti-infection effects of *B. amyloliquefaciens* against pathogenic fungi causing diseases in rice, tomato, potato, brinjal and fruit (Jiao et al., 2020; Nie et al., 2022; Marković et al., 2023; Pradhan et al., 2023; Zhang et al., 2023). *B. amyloliquefaciens* has demonstrated potent capabilities in crop disease control, but at present, it is only reported that *B. amyloliquefaciens* can promote the root growth of replanted *Malus hupehensis* Rehd. seedlings that is a rootstock variety of apple, and it is still necessary to explore new strains of *B. amyloliquefaciens* for the control of ARD on different rootstock varieties.

In this study, we successfully demonstrated the effectiveness of the *B. amyloliquefaciens* strain BV-4 in mitigating the prevalence of ARD on *Malus robusta* Rehd. seedlings, which is the most common apple rootstock variety in northern China, but unfortunately it does not have the resistance to ARD. Strain BV-4 not only reduces the incidence of ARD induced by *Fusarium* species but also alleviates the adverse impact it exerts on the growth of MR seedlings. Furthermore, our research sheds light on the broad-spectrum antagonistic activity exhibited by the BA-4 strain against various plant pathogens, many of the reported *Bacillus* strains that have been identified also exhibit similarly outstanding antagonistic activity by producing antimicrobial substances (Fira et al., 2018; Chen et al., 2020; Etesami et al., 2023). This characteristic positions BA-4 as a versatile and potent tool in the arsenal against not only *Fusarium*-induced ARD but also a spectrum of other plant fungal diseases.

Various biocontrol mechanisms have been described for *B. amyloliquefaciens* as a biocontrol agent against soil-borne fungal diseases (Fira et al., 2018; Etesami et al., 2023). Among the most important strategies is the efficient synthesis of various substances with antimicrobial activity. Following the antagonistic interaction with the *B. amyloliquefaciens* strain BA-4, the hyphae of ARD-related *Fusarium* pathogens exhibited abnormal elongation, increased branching, shortened morphology, thin deformation, and cytoplasmic-like spheres at the hyphal tips. Similar reaction had been reported in the interaction between *F. chlamydosporum*, the pathogenic agent responsible for stem rot in *Jacaranda acutifolia*, and *B. amyloliquefaciens*. This reaction is mainly caused by antimicrobial peptides produced by *B. amyloliquefaciens* in fermentation broth (Zhu and Pan, 2019). Further investigation indicates that the cell-free culture filtrate of the BA-4 strain demonstrated effective antifungal activity against ARD-related *Fusarium* pathogens and inhibited spore germination, which led us to speculate that the strain BA-4 synthesizes antimicrobial compounds and secretes them into the extracellular supernatant. Typically, *Bacillus* species possessed gene clusters within their genomes engaged in the synthesis of antifungal lipopeptides and polyketides (Aleti et al., 2015; Caulier et al., 2019; Fazle Rabbee and Baek, 2020). In the case of *B. amyloliquefaciens* FZB42 harbors multiple gene clusters dedicated to the synthesis of secondary metabolites with antifungal and antibacterial properties. These include non-ribosomal synthesis of cyclic lipopeptides such as surfactin, bacillomycin, fengycin, an unidentified peptide, and the iron siderophore bacillibactin. This synthesis of secondary metabolites is essential for effectively managing and responding to other competitive pathogenic fungi present in the plant rhizosphere (Chen et al., 2008; Chowdhury et al., 2015). Therefore, the capacity to generate these metabolites containing anti-fungal active compounds constitutes a crucial mechanism contributing to the functionality of the *Bacillus* agent.

In addition to the potential for producing antifungal metabolites, *B. amyloliquefaciens* strain BA-4 also secretes lytic enzymes such as protease, cellulose, as well as produced siderophores which significantly disrupt the structural integrity of fungal cell walls. The fundamental structural components of the cell wall in phytopathogenic fungi are chitin and proteins, crucial for pathogenesis and disease transmission (Kong et al., 2012; Ma et al., 2015). Other, microbially-produced cellulase can serve as an elicitor to stimulate plant immunity during interactions between plants and pathogens (Jha et al., 2010; Chakraborty et al., 2020). Siderophores, high-affinity iron-chelating compounds secreted by beneficial bacteria, exhibit strong binding affinity to ferric ions and have the potential to hinder the growth of pathogenic fungi by limiting the availability of iron essential for their normal metabolic processes (Carmona-Hernandez et al., 2019; Pradhan et al., 2023). Consequently, the production of protease, cellulose and siderophore by BA-4 may contribute to the distortion of structural integrity of ARD-related *Fusarium* pathogens, resulting in mycelial deformation and abnormal sporulation, interfering with the normal metabolism of these pathogens, triggering the immune response in plant seedlings. These findings provide additional essential evidence supporting the effective inhibition of pathogenic fungi by *B. amyloliquefaciens* strain BA-4.

In this study, strain BA-4 exhibited a variety of characteristics associated with promoting plant growth, including the synthesis of siderophores known for their role in enhancing plant growth under iron-limiting conditions (Radzki et al., 2013; Kumar et al., 2017). These characteristics encompassed activities such as phosphate solubilization, potassium solubilization, and the IAA production. The phosphate and potassium-solubilizing microorganisms play a key driving force enabling higher absorption of P and K by secreting organic acids and excreting siderophores, which chelate metal ions and form complexes, making phosphates available for plant uptake (Kashyap et al., 2021; Jain et al., 2022). These microorganisms not only solubilize phosphate and potassium but also promote plant growth and crop yield by producing plant-growth-promoting hormones like auxins, thereby fostering robust plant growth and root development (Saxena et al., 2020). Consistent with this, our results demonstrated that the treatment with the BA-4 strain significantly enhanced the root activity of MR seedlings. These multifaceted plant growth promotion traits suggest that the strain BA-4 not only contributes to pathogen suppression but also actively supports overall plant growth through various beneficial mechanisms.

Rhizospheric microorganisms are particularly well-suited for deployment as biological controls against soil-borne pathogens, as the rhizosphere provides a bleeding edge resistance to roots against pathogenic assaults, and it is also crucial for establishing an effective root system configuration to support optimal plant growth (Singh and Sachdev, 2018; Middleton et al., 2021). A study conducted by Duan et al. (2021) demonstrated that the application of *B. amyloliquefaciens* strain QSB-6, isolated from rhizosphere soil, significantly enhanced the growth of *Malus hupehensis* Rehd. seedling in pot experiments. This improvement was observed in increased root length, surface area, tips, and forks, and effectively reduced the abundance of soil fungi and prevented root damage from ARD pathogens. In our study, we examined the effectiveness of the rhizosphere bacterium *B. amyloliquefaciens* BA-4 in mitigating ARD symptoms associated with *Fusarium* through visual inspection of MR seedlings in the field. Plant height and other growth indicators representing the disease intensity significantly improved after BA-4 treatment. Additionally, another study revealed that a microbial fertilizer containing *Bacillus* sp. could alleviate ARD symptoms by altering the microbial community structure in rhizosphere soil (Geng et al., 2022). This suggests that the application of BA-4 may play a crucial role in improving the rhizosphere microbial community, subsequently affecting plant growth, and resistance to soil-borne pathogens.

Microbial coexistence in the rhizosphere play a crucial role in aiding plants withstand both abiotic and biotic stresses, offering opportunities to enhance agricultural production sustainability and plant disease resistance (Ghoul and Mitri, 2016; Bennett and Klironomos, 2019). As an apple tree is a perennial woody plant, the accumulation of ARD-related pathogenic fungi in the rhizosphere increases over the years of apple cultivation. Consequently, swiftly and effectively addressing *Fusarium* pathogens poses a significant challenge. Numerous studies focus on identifying BCAs with long-term soil or root-colonizing capabilities for inhibiting or coexisting with ARD-related pathogens while maintaining the pathogens below the critical threshold. For instance, the strain QSB-6 of *B. amyloliquefaciens* showcased the capability to colonize the infection site of *Malus hupehensis* Rehd. seedling, forming a protective biofilm on the root epidermis to ward off intrusion by ARD pathogens. Additionally, the *B. licheniformis* XNRB-3, isolated from root tissues, exhibited enduring colonization on the roots of apple seedlings. The introduction of the XNRB-3 strain may have a prolonged growth-promoting and biocontrol influence on apple plants confronted with the challenges of ARD (Duan et al., 2022b). In this study, the colonization ability of *B. amyloliquefaciens* BA-4 in MR seedling rhizosphere and its biocontrol effect on ARD were evaluated in the field to verify the findings obtained from pot experiments. The strain BA-4 demonstrated the capability to maintain a population level of 10^5^ CFU/g in rhizosphere of MR seedling. The evaluation conducted on the 90th day after planting revealed that the treatment with strain BA-4 significantly reduced the mortality rate of apple plants caused by ARD. Hence, we speculate that the capacity of *B. amyloliquefaciens* BA-4 to stimulate growth and suppress *Fusarium* pathogens is intricately linked to its colonization ability. A consistent and stable presence in the apple rhizosphere proves particularly advantageous for strain BA-4 to effectively carry out its role in biocontrol.

Numerous plant phytopathogens, including *Fusarium* spp., have exhibited resistance to fungicides such as carbendazim, Benzimidazoles and fludioxonil (Duan et al., 2019; Zhou et al., 2020), which is one of the emerging challenges in plant disease management strategies. These findings encouraged us to emphasize the efficiency of BCAs in controlling preventing fungicide resistance in *Fusarium* isolates. Moreover, we found that *B. amyloliquefaciens* BA-4 does not exhibit antagonistic effects with other commonly used biocontrol microorganisms such as other *Bacillus*, *Pseudomonas*, and *Trichoderma* strains (unpublish data). This absence of antagonism suggests that BA-4 can coexist compatibly with these potential future commercialized biocontrol agents. Whether used in rotation or combination with these microorganisms, BA-4 not only minimizes the risk of decreased efficacy but also alleviates the threat of resistance development associated with the prolonged use of a singular biocontrol agent. Given that biocontrol *Bacillus* spp. mainly function by secreting antibiotic-like substances, the potential risks associated with pathogen resistance and the transfer of resistance genes between biocontrol agents and pathogenic bacteria are also problems worthy of attention. Nevertheless, the utilization of BA-4, either alone or in conjunction with other agents, contributes to the sustainable control of ARD, making it a preferred option to ensure high and stable apple yields in the future.

In conclusion, *B. amyloliquefaciens* BA-4, isolated from the rhizosphere of healthy apple trees, holds promise as a prospective BCA and growth enhancer for the management of soil-borne diseases in apple cultivation. It offers an eco-friendly and sustainable alternative to traditional fungicides, aiding in apple yield improvement. The stable colonization and the production of active metabolites by BA-4 may serve as a potent strategy to reduce ARD infections, providing a natural substitute for chemically synthesized pesticides harmful to the environment. Future research endeavors should focus on evaluating the biocontrol efficacy of strain BA-4 against *Fusarium*-related ARD of different *Malus* rootstock varieties in multiple locations and seasons. Additionally, optimizing the fermentation conditions of strain BA-4 is crucial for enhancing their antagonistic efficiency against plant pathogens and facilitating their commercialization in field applications.

## Data availability statement

The original contributions presented in the study are included in the article/supplementary material. Further inquiries can be directed to the corresponding author.

## Author contributions

BL: Methodology, Writing – original draft, Writing – review & editing. XH: Investigation, Visualization, Writing – review & editing. SG: Investigation, Writing – review & editing. DL: Investigation, Writing – review & editing. YW: Writing – review & editing. XM: Writing – review & editing. PD: Writing – review & editing. TH: Supervision, Writing – review & editing. KC: Funding acquisition, Project administration, Supervision, Writing – review & editing. SW: Funding acquisition, Project administration, Supervision, Writing – review & editing.

## References

[B1] Ajeethan N. Ali S. Fuller K. D. Abbey L. Yurgel S. N. (2023). Apple root microbiome as indicator of plant adaptation to apple replant diseased soils. Microorganisms 11, 1372. doi: 10.3390/microorganisms11061372 37374874 PMC10301482

[B2] Aleti G. Sessitsch A. Brader G. (2015). Genome mining: Prediction of lipopeptides and polyketides from *Bacillus* and related Firmicutes. Comput. Struct. Biotechnol. J. 13, 192–203. doi: 10.1016/j.csbj.2015.03.003 25893081 PMC4397504

[B3] Bais H. P. Fall R. Vivanco J. M. (2004). Biocontrol of *Bacillus subtilis* against infection of arabidopsis roots by *Pseudomonas syringae* is facilitated by biofilm formation and surfactin production. Plant Physiol. 134, 307–319. doi: 10.1104/pp.103.028712 14684838 PMC316310

[B4] Bennett J. A. Klironomos J. (2019). Mechanisms of plant–soil feedback: interactions among biotic and abiotic drivers. New Phytol. 222, 91–96. doi: 10.1111/nph.15603 30451287

[B5] Carmona-Hernandez S. Reyes-Pérez J. Chiquito-Contreras R. Rincon-Enriquez G. Cerdan-Cabrera C. Hernandez-Montiel L. (2019). Biocontrol of postharvest fruit fungal diseases by bacterial antagonists: A Review. Agronomy 9, 121. doi: 10.3390/agronomy9030121

[B6] Caulier S. Nannan C. Gillis A. Licciardi F. Bragard C. Mahillon J. (2019). Overview of the antimicrobial compounds produced by members of the *Bacillus subtilis* group. Front. Microbiol. 10. doi: 10.3389/fmicb.2019.00302 PMC6401651 30873135

[B7] Cavael U. Lentzsch P. Schwärzel H. Eulenstein F. Tauschke M. Diehl K. (2021). Assessment of agro-ecological apple replant disease (ARD) management strategies: Organic fertilization and inoculation with mycorrhizal fungi and bacteria. Agronomy 11 (2), 272. doi: 10.3390/agronomy11020272

[B8] Cheffi Azabou M. Gharbi Y. Medhioub I. Ennouri K. Barham H. Tounsi S.. (2020). The endophytic strain *Bacillus velezensis* OEE1: An efficient biocontrol agent against *Verticillium* wilt of olive and a potential plant growth promoting bacteria. Biol. Control 142, 104168. doi: 10.1016/j.biocontrol.2019.104168

[B9] Chen X. H. Koumoutsi A. Scholz R. Borriss R. (2008). More than anticipated - Production of antibiotics and other secondary metabolites by *Bacillus amyloliquefaciens* FZB42. J. Mol. Microbiol. Biotechnol. 16, 14–24. doi: 10.1159/000142891 18957859

[B10] Chen Q. Qiu Y. Yuan Y. Wang K. Wang H. (2022). Biocontrol activity and action mechanism of *Bacillus velezensis* strain SDTB038 against *Fusarium* crown and root rot of tomato. Front. Microbiol. 13. doi: 10.3389/fmicb.2022.994716 PMC9479544 36118232

[B11] Chen K. Tian Z. He H. Long C. Jiang F. (2020). *Bacillus* species as potential biocontrol agents against citrus diseases. Biol. Control 151, 104419. doi: 10.1016/j.biocontrol.2020.104419

[B12] Chowdhury S. P. Uhl J. Grosch R. Alquéres S. Pittroff S. Dietel K.. (2015). Cyclic lipopeptides of *Bacillus amyloliquefaciens* subsp. *plantarum* colonizing the lettuce rhizosphere enhance plant defense responses toward the bottom rot pathogen *Rhizoctonia solani* . Mol. Plant-Microbe Interact. 28, 984–995. doi: 10.1094/MPMI-03-15-0066-R 26011557

[B13] Crutcher F. K. Puckhaber L. S. Stipanovic R. D. Bell A. A. Nichols R. L. Lawrence K. S.. (2017). Microbial resistance mechanisms to the antibiotic and phytotoxin fusaric acid. J. Chem. Ecol. 43, 996–1006. doi: 10.1007/s10886-017-0889-x 28986689

[B14] Cui W. He P. Munir S. He P. Li X. Li Y.. (2019). Efficacy of plant growth promoting bacteria *Bacillus amyloliquefaciens* B9601-Y2 for biocontrol of southern corn leaf blight. Biol. Control 139, 104080. doi: 10.1016/j.biocontrol.2019.104080

[B15] Ding T. Su B. Chen X. Xie S. Gu S. Wang Q.. (2017). An endophytic bacterial strain isolated from *Eucommia ulmoides* inhibits southern corn leaf blight. Front. Microbiol. 8, 00903. doi: 10.3389/fmicb.2017.00903 PMC5435801 28572799

[B16] Dong X. Ling N. Wang M. Shen Q. Guo S. (2012). Fusaric acid is a crucial factor in the disturbance of leaf water imbalance in *Fusarium*-infected banana plants. Plant Physiol. Biochem. 60, 171–179. doi: 10.1016/j.plaphy.2012.08.004 22964424

[B17] Duan Y. Chen R. Zhang R. Jiang W. Chen X. Yin C.. (2021). Isolation, identification, and antibacterial mechanisms of *Bacillus amyloliquefaciens* QSB-6 and its effect on plant roots. Front. Microbiol. 12. doi: 10.3389/fmicb.2021.746799 PMC8482014 34603274

[B18] Duan Y. Chen R. Zhang R. Jiang W. Chen X. Yin C.. (2022a). Isolation and identification of *Bacillus vallismortis* HSB-2 and its biocontrol potential against apple replant disease. Biol. Control 170, 104921. doi: 10.1016/j.biocontrol.2022.104921

[B19] Duan Y. N. Ma S. R. Chen X. S. Shen X. Yin C. M. Mao Z. Q. (2023). Genome sequence resource of *Fusarium proliferatum* f. sp. *malus domestica* MR5, the causative agent of apple replant disease. Plant Dis. 107, 903–907. doi: 10.1094/PDIS-06-22-1352-A 36587236

[B20] Duan Y. Tao X. Zhao H. Xiao X. Li M. Wang J.. (2019). Activity of demethylation inhibitor fungicide metconazole on Chinese *Fusarium graminearum* species complex and its application in carbendazim-resistance management of *Fusarium* head blight in wheat. Plant Dis. 103, 929–937. doi: 10.1094/PDIS-09-18-1592-RE 30880557

[B21] Duan Y. Zhao L. Jiang W. Chen R. Zhang R. Chen X.. (2022b). The Phlorizin-Degrading *Bacillus licheniformis* XNRB-3 mediates soil microorganisms to alleviate apple replant disease. Front. Microbiol. 13. doi: 10.3389/fmicb.2022.839484 PMC8927668 35308362

[B22] Etesami H. Jeong B. R. Glick B. R. (2023). Biocontrol of plant diseases by *Bacillus* spp. Physiol. Mol. Plant Pathol. 126, 102048. doi: 10.1016/j.pmpp.2023.102048

[B23] Fan H. He P. Xu S. Li S. Wang Y. Zhang W.. (2023). Banana disease-suppressive soil drives *Bacillus* assembled to defense *Fusarium* wilt of banana. Front. Microbiol. 14. doi: 10.3389/fmicb.2023.1211301 PMC10437119 37601384

[B24] Fazle Rabbee M. Baek K. H. (2020). Antimicrobial activities of lipopeptides and polyketides of *Bacillus velezensis* for agricultural applications. Molecules 25 (21), 4973. doi: 10.3390/molecules25214973 33121115 PMC7662345

[B25] Fira D. Dimkić I. Berić T. Lozo J. Stanković S. (2018). Biological control of plant pathogens by *Bacillus* species. J. Biotechnol. 285, 44–55. doi: 10.1016/j.jbiotec.2018.07.044 30172784

[B26] Geng W. Lv Y. Duan Y. Wang H. Jiang W. Zhang R.. (2022). Preparation of composite microbial culture and its biocontrol effect on apple replant disease. Sci. Hortic. (Amsterdam) 303, 111236. doi: 10.1016/j.scienta.2022.111236

[B27] Ghoul M. Mitri S. (2016). The ecology and evolution of microbial competition. Trends Microbiol. 24, 833–845. doi: 10.1016/j.tim.2016.06.011 27546832

[B28] Höfte M. (2021). The use of Pseudomonas spp. as bacterial biocontrol agents to control plant diseases. In Köhl J. Ravensberg W. J. (Eds.), Microbial bioprotectants for plant disease management. 301–374. doi: 10.19103/AS.2021.0093.11

[B29] Jain D. Saheewala H. Sanadhaya S. Joshi A. Bhojiya A. A. Verma A. K.. (2022). “Potassium solubilizing microorganisms as soil health engineers: An insight into molecular mechanism,” in Rhizosphere Engineering (Elsevier), 199–214. doi: 10.1016/B978-0-323-89973-4.00007-7

[B30] Jangir M. Pathak R. Sharma S. Sharma S. (2018). Biocontrol mechanisms of *Bacillus* sp., isolated from tomato rhizosphere, against *Fusarium oxysporum* f. sp. *lycopersici* . Biol. Control 123, 60–70. doi: 10.1016/j.biocontrol.2018.04.018

[B31] Jia Q. Fan Y. Duan S. Qin Q. Ding Y. Yang M.. (2023). Effects of *Bacillus amyloliquefaciens* XJ-BV2007 on growth of *Alternaria alternata* and production of tenuazonic acid. Toxins (Basel) 15 (1), 53. doi: 10.3390/toxins15010053 36668873 PMC9867350

[B32] Jiang W. Chen R. Zhao L. Qin L. Fan H. Chen X.. (2022). Chemical fumigants control apple replant disease: Microbial community structure-mediated inhibition of *Fusarium* and degradation of phenolic acids. J. Hazard. Mater. 440, 129786. doi: 10.1016/j.jhazmat.2022.129786 36007363

[B33] Jiao R. Munir S. He P. Yang H. Wu Y. Wang J.. (2020). Biocontrol potential of the endophytic *Bacillus amyloliquefaciens* YN201732 against tobacco powdery mildew and its growth promotion. Biol. Control 143, 104160. doi: 10.1016/j.biocontrol.2019.104160

[B34] Kanfra X. Wrede A. Mahnkopp-Dirks F. Winkelmann T. Heuer H. (2022). Networks of free-living nematodes and co-extracted fungi, associated with symptoms of apple replant disease. Appl. Soil Ecol. 172, 104368. doi: 10.1016/j.apsoil.2021.104368

[B35] Kashyap A. S. Manzar N. Nebapure S. M. Rajawat M. V. S. Deo M. M. Singh J. P.. (2022). Unraveling microbial volatile elicitors using a transparent methodology for induction of systemic resistance and regulation of antioxidant genes at expression levels in Chili against Bacterial Wilt Disease. Antioxidants 11 (2), 404. doi: 10.3390/antiox11020404 35204287 PMC8869530

[B36] Kashyap A. S. Manzar N. Rajawat M. V. S. Kesharwani A. K. Singh R. P. Dubey S. C.. (2021). Screening and biocontrol potential of rhizobacteria native to gangetic plains and Hilly regions to induce systemic resistance and promote plant growth in Chilli against Bacterial Wilt Disease. Plants 10, 2125. doi: 10.3390/plants10102125 34685934 PMC8541367

[B37] Kelderer M. Manici L. M. Caputo F. Thalheimer M. (2012). Planting in the “inter-row” to overcome replant disease in apple orchards: A study on the effectiveness of the practice based on microbial indicators. Plant Soil 357, 381–393. doi: 10.1007/s11104-012-1172-0

[B38] Khan N. Martínez-Hidalgo P. Ice T. A. Maymon M. Humm E. A. Nejat N.. (2018). Antifungal activity of *Bacillus* species against *Fusarium* and analysis of the potential mechanisms used in biocontrol. Front. Microbiol. 9, 02363. doi: 10.3389/fmicb.2018.02363 PMC6176115 30333816

[B39] Kong L.-A. Yang J. Li G.-T. Qi L.-L. Zhang Y.-J. Wang C.-F.. (2012). Different chitin synthase genes are required for various developmental and plant infection processes in the rice blast fungus *Magnaporthe oryzae* . PloS Pathog. 8, e1002526. doi: 10.1371/journal.ppat.1002526 22346755 PMC3276572

[B40] Krieg N. R. Staley J. T. Brown D. R. Hedlund B. P. Paster B. J. Ward N. L.. eds. (2010). Bergey’s Manual® of Systematic Bacteriology. New York, NY: Springer New York. doi: 10.1007/978-0-387-68572-4

[B41] Kumar V. Kumar M. Sharma S. Prasad R. (2017). Probiotics and Plant Health. Eds. Kumar V. Kumar M. Sharma S. Prasad R. (Singapore: Springer Singapore). doi: 10.1007/978-981-10-3473-2

[B42] Li B. Kong L. Qiu D. Francis F. Wang S. (2021). Biocontrol potential and mode of action of entomopathogenic bacteria *Xenorhabdus budapestensis* C72 against *Bipolaris maydis* . Biol. Control 158, 104605. doi: 10.1016/j.biocontrol.2021.104605

[B43] Liu X. Xu S. Wang X. Xin L. Wang L. Mao Z.. (2022). MdBAK1 overexpression in apple enhanced resistance to replant disease as well as to the causative pathogen *Fusarium oxysporum* . Plant Physiol. Biochem. 179, 144–157. doi: 10.1016/j.plaphy.2022.03.014 35344759

[B44] Ma Y. Han C. Chen J. Li H. He K. Liu A.. (2015). Fungal cellulase is an elicitor but its enzymatic activity is not required for its elicitor activity. Mol. Plant Pathol. 16, 14–26. doi: 10.1111/mpp.12156 24844544 PMC6638370

[B45] Mahnkopp F. Simon M. Lehndorff E. Pätzold S. Wrede A. Winkelmann T. (2018). Induction and diagnosis of apple replant disease (ARD): a matter of heterogeneous soil properties? Sci. Hortic. (Amsterdam) 241, 167–177. doi: 10.1016/j.scienta.2018.06.076

[B46] Manici L. M. Caboni E. Caputo F. Frattarelli A. Lucioli S. (2021). Phytotoxins from Dactylonectria torresensis involved in replant disease of fruit trees. Rhizosphere 17, 100300. doi: 10.1016/j.rhisph.2020.100300

[B47] Manici L. M. Kelderer M. Caputo F. Saccà M. L. Nicoletti F. Topp A. R.. (2018). Involvement of *Dactylonectria* and Ilyonectria spp. in tree decline affecting multi-generation apple orchards. Plant Soil, 425. doi: 10.1007/s11104-018-3571-3

[B48] Manzar N. Kashyap A. S. Goutam R. S. Rajawat M. V. S. Sharma P. K. Sharma S. K.. (2022). Trichoderma: advent of versatile biocontrol agent, its secrets and insights into mechanism of biocontrol potential. Sustainability (Switzerland) 14 (19), 12786. doi: 10.3390/su141912786

[B49] Marković S. Milovanović T. P. Jelušić A. Iličić R. Medić O. Berić T.. (2023). Biological control of major pathogenic bacteria of potato by *Bacillus amyloliquefaciens* strains SS-12.6 and SS-38.4. Biol. Control 182, 105238. doi: 10.1016/j.biocontrol.2023.105238

[B50] Massart S. Margarita M. M. Jijakli M. H. (2015). Biological control in the microbiome era: Challenges and opportunities. Biol. Control 89, 98–108. doi: 10.1016/j.biocontrol.2015.06.003

[B51] Middleton H. Yergeau É. Monard C. Combier J. P. El Amrani A. (2021). Rhizospheric plant–microbe interactions: miRNAs as a key mediator. Trends Plant Sci. 26, 132–141. doi: 10.1016/j.tplants.2020.09.005 33036916

[B52] Nicola L. Turco E. Albanese D. Donati C. Thalheimer M. Pindo M.. (2017). Fumigation with dazomet modifies soil microbiota in apple orchards affected by replant disease. Appl. Soil Ecol. 113, 71–79. doi: 10.1016/j.apsoil.2017.02.002

[B53] Nie L. J. Ye W. Q. Xie W. Y. Zhou W. W. (2022). Biofilm: New insights in the biological control of fruits with *Bacillus amyloliquefaciens* B4. Microbiol. Res. 265, 127196. doi: 10.1016/j.micres.2022.127196 36116146

[B54] Poulaki E. G. Tjamos S. E. (2023). *Bacillus* species: factories of plant protective volatile organic compounds. J. Appl. Microbiol. 134 (3), lxad037. doi: 10.1093/jambio/lxad037 36822621

[B55] Pradhan S. Choudhury A. Dey S. Hossain M. F. Saha A. Saha D. (2023). Siderophore-producing *Bacillus amyloliquefaciens* BM3 mitigate arsenic contamination and suppress Fusarium wilt in brinjal plants. J. Appl. Microbiol. 134, 1–13. doi: 10.1093/jambio/lxad217 37740438

[B56] Qiao J. Zhang R. Liu Y. Liu Y. (2023). Evaluation of the biocontrol efficiency of *Bacillus subtilis* wettable powder on pepper root rot caused by *Fusarium solani* . Pathogens 12. doi: 10.3390/pathogens12020225 PMC9967462 36839497

[B57] Radzki W. Gutierrez Mañero F. J. Algar E. Lucas García J. A. García-Villaraco A. Ramos Solano B. (2013). Bacterial siderophores efficiently provide iron to iron-starved tomato plants in hydroponics culture. Antonie van Leeuwenhoek Int. J. Gen. Mol. Microbiol. 104, 321–330. doi: 10.1007/s10482-013-9954-9 PMC3739868 23812968

[B58] Raymaekers K. Ponet L. Holtappels D. Berckmans B. Cammue B. P. A. (2020). Screening for novel biocontrol agents applicable in plant disease management – A review. Biol. Control 144, 104240. doi: 10.1016/j.biocontrol.2020.104240

[B59] Reyes-Estebanez M. Sanmartín P. Camacho-Chab J. C. de la Rosa-García S. C. Chan-Bacab M. J. Águila-Ramírez R. N.. (2020). Characterization of a native *Bacillus velezensis*-like strain for the potential biocontrol of tropical fruit pathogens. Biol. Control 141, 104127. doi: 10.1016/j.biocontrol.2019.104127

[B60] Sarangi T. Ramakrishnan S. (2023). Biocontrol potential and mechanism of *Bacillus* spp. against phytopathogens: A Review. Int. J. Environ. Clim. Change 13, 512–527. doi: 10.9734/ijecc/2023/v13i71904

[B61] Saxena A. K. Kumar M. Chakdar H. Anuroopa N. Bagyaraj D. J. (2020). *Bacillus* species in soil as a natural resource for plant health and nutrition. J. Appl. Microbiol. 128, 1583–1594. doi: 10.1111/jam.14506 31705597

[B62] Shafi J. Tian H. Ji M. (2017). *Bacillus* species as versatile weapons for plant pathogens: a review. Biotechnol. Biotechnol. Equip. 31, 446–459. doi: 10.1080/13102818.2017.1286950

[B63] Singh R. P. Sachdev S. (2018). Root colonization: imperative mechanism for efficient plant protection and growth. MOJ Ecol. Environ. Sci. 3, 240–242. doi: 10.15406/mojes.2018.03.00094

[B64] Somera T. S. Mazzola M. (2022). Toward a holistic view of orchard ecosystem dynamics: A comprehensive review of the multiple factors governing development or suppression of apple replant disease. Front. Microbiol. 13. doi: 10.3389/fmicb.2022.949404 PMC9358454 35958152

[B65] Vurukonda S. S. K. P. Giovanardi D. Stefani E. (2018). Plant growth promoting and biocontrol activity of *Streptomyces* spp. as endophytes. Int. J. Mol. Sci. 19, 952. doi: 10.3390/ijms19040952 29565834 PMC5979581

[B66] Wang H. Tang W. Mao Y. Ma S. Chen X. Shen X.. (2022a). Isolation of *Trichoderma virens* 6PS-2 and its effects on *Fusarium proliferatum* f. sp. *Malus domestica* MR5 related to apple replant disease (ARD) in China. Hortic. Plant J. doi: 10.1016/j.hpj.2022.09.007

[B67] Wang X. Yao Y. Wang G. Lu H. Ma J. Zhang M.. (2022b). Controlled-release diammonium phosphate alleviates apple replant disease: An integrated analysis of soil properties, plant growth, and the soil microbiome. J. Agric. Food Chem. 70, 8942–8954. doi: 10.1021/acs.jafc.2c01630 35835727

[B68] Winkelmann T. Smalla K. Amelung W. Baab G. Grunewaldt-Stöcker G. Kanfra X.. (2019). Apple replant disease: Causes and mitigation strategies. Curr. Issues Mol. Biol. 30, 89–106. doi: 10.21775/cimb.030.089 30070653

[B69] Wu Y. Zhou J. Li C. Ma Y. (2019). Antifungal and plant growth promotion activity of volatile organic compounds produced by *Bacillus amyloliquefaciens* . Microbiologyopen 8. doi: 10.1002/mbo3.813 PMC6692555 30907064

[B70] Xiang L. Wang M. Jiang W. Wang Y. Chen X. Yin C.. (2021a). Key indicators for renewal and reconstruction of perennial trees soil: Microorganisms and phloridzin. Ecotoxicol Environ. Saf 225. doi: 10.1016/j.ecoenv.2021.112723 34481354

[B71] Xiang L. Zhao L. Wang M. Huang J. Chen X. Yin C.. (2021b). Physiological responses of apple rootstock M.9 to infection by *Fusarium solani* . HortScience 59, 1104–1111. doi: 10.21273/HORTSCI15945-21

[B72] Yan Y. Xu W. Hu Y. Tian R. Wang Z. (2022). *Bacillus velezensis* YYC promotes tomato growth and induces resistance against bacterial wilt. Biol. Control 172, 104977. doi: 10.1016/j.biocontrol.2022.104977

[B73] Zhang H. Zhang R. Qiao J. Yu J. Qi Z. Du Y.. (2023). Optimization of *Bacillus amyloliquefaciens* Lx-11 suspoemulsion by response surface methodology to control rice bacterial blight. BioControl 68, 169–179. doi: 10.1007/s10526-023-10186-6

[B74] Zheng Y. Wang X. Liu S. Zhang K. Cai Z. Chen X.. (2018). The endochitinase of *Clonostachysrosea* expression in *Bacillus amyloliquefaciens* enhances the Botrytis cinerea resistance of tomato. Int. J. Mol. Sci. 19, 2221. doi: 10.3390/ijms19082221 30061502 PMC6121428

[B75] Zhou F. Li D. X. Hu H. Y. Song Y. L. Fan Y. C. Guan Y. Y.. (2020). Biological characteristics and molecular mechanisms of fludioxonil resistance in *Fusarium graminearum* in China. Plant Dis. 104, 2426–2433. doi: 10.1094/PDIS-01-20-0079-RE 32658633

[B76] Zhu H. M. Pan Y.-Z. (2019). A novel antimicrobial protein of the endophytic Bacillus amyloliquefaciens and its control effect against *Fusarium chlamydosporum* . BioControl 64, 737–748. doi: 10.1007/s10526-019-09972-y

